# High number of hospitalisations and non-classical presentations: lessons learned from a measles outbreak in 2017, Belgium

**DOI:** 10.1017/S0950268820000278

**Published:** 2020-02-24

**Authors:** L. Cornelissen, T. Grammens, S. Leenen, C. Schirvel, V. Hutse, R. Demeester, B. Swennen, T. Asikainen, C. Wyndham-Thomas

**Affiliations:** 1 Service of Epidemiology of Infectious Diseases, Department of Epidemiology and Public Health, Sciensano, Brussels, Belgium; 2 Infectious Disease Surveillance Unit, Agence pour une Vie de Qualité (AVIQ), Walloon region, Charleroi, Belgium; 3 National Reference Centre for Measles, Mumps and Rubella, Service of Viral Diseases, Sciensano, Brussels, Belgium; 4 Centre Hospitalier Universitaire de Charleroi, Charleroi, Belgium; 5 Public Health School - Université Libre de Bruxelles, Brussels, Belgium

**Keywords:** Epidemiology, measles (rubeola), notifiable infectious diseases, occupation-related infections, vaccine preventable diseases

## Abstract

We describe and analyse an outbreak of measles that affected Belgium early 2017. In total, 289 cases were reported, mostly (53%) in people 15 years or older. For 133 (46%) vaccination status was unknown and a further 117 (41%) were not vaccinated. According to national guidelines, 83 of the unvaccinated cases (29% of total cases) should have received minimum one dose of vaccine, but did not. One in five cases (21%) did not present with the classical triad of fever, rash and any of coryza, conjunctivitis or cough. Rash was the most sensitive symptom, being absent in only six cases. A large proportion of cases (125/289, 43%) required hospitalisation. In hospitalised patients, the most commonly observed complications were hepatic disorders (present in 58/125 hospitalised patients, 46%). Thirty-six of the cases (12%) were in healthcare workers and nosocomial spread contributed importantly to the outbreak. Older age at presentation, altered clinical presentations and presence of complications like hepatitis can delay the correct diagnosis of measles. Clinicians should maintain a high index of suspicion in any individual presenting with rash. If the elimination target is to be reached, catch-up vaccination campaigns should be intensified and target young adults and health care workers.

## Introduction

Measles is a highly contagious disease caused by a *morbillivirus* and transmitted from person to person via respiratory droplets or contaminated surfaces. A typical case of measles is defined by the presence of fever in combination with maculo-papular rash and either cough, coryza or conjunctivitis [1]. It is associated with non-negligible morbidity and mortality, as typically 10 up to 30% of cases present with complications and case fatality rates lie between 1 and 3 per 1000 cases [2 3].

In Belgium, measles vaccine is administered in the live-attenuated combination vaccine of Measles–Mumps–Rubella (MMR) and has been offered free of charge in the routine immunisation programme since 1985. It is 93% effective against measles after a single dose (MMR1 offered in Belgium at 12 months) and 97% effective after two-doses (MMR2 offered at 10–12 years) [4]. People born before 1970 are considered immune due to exposure to then widely circulating measles. Vaccination coverage in children has been above 95% for the first dose of MMR vaccine since 2015, but remains lower for the second-dose of MMR, estimated in 2016 at 75% for the Brussels and Wallonia regions [5].

Child-vaccination programmes with MMR have induced a massive reduction in the worldwide incidence of measles and measles-related deaths [6]. In 2012, the World Health Assembly pledged to eliminate measles and rubella in at least 5 WHO regions by 2020 [7]. Nevertheless, outbreaks continue to arise across the globe and in 2017 the EU/EEA saw 14 600 persons affected by measles and 37 reported measles deaths [8]. In Belgium, measles cases are under mandatory reporting to regional health authorities. In 2017, it was one of the four countries in the European region still considered endemic for measles. The last large-scale outbreak in the country dated back to 2011 and since 2013 the annual measles-incidence had been oscillating between 3.5 and 6.3 cases per million inhabitants [9].

Here we describe an outbreak that affected the Wallonia region in 2017. A brief report was published previously [10]. In this article, we provide a description of the outbreak as well as a detailed analysis of clinical aspects like non-classical presentations, hospitalisations and complications.

## Methods

### Case definition and detection

Cases were identified either through mandatory notifications to or contact tracing by Wallonia's Infectious Disease Surveillance Unit (AViQ). Measles cases were defined as ‘possible’ (clinical case), ‘probable’ (clinical case with an epidemiological link) or ‘confirmed’ (clinical case confirmed by lab testing) based on the ECDC definition as published in the European Union (EU) Commission Decision of 2012 [1]. Patients not meeting the case definition but with a clinical suspicion in combination with either an epidemiological link or laboratory confirmation, were also considered as probable or confirmed cases, respectively.

### Outbreak containment

Prophylactic vaccination against measles was recommended to all persons who had contact with a measles case less than 72 h before and who were not considered immune or did not know their vaccination status. Large information campaigns for the broad public were released through the general press. Schools and child care facilities were informed by targeted campaigns and health professionals received tailored information and reminders.

### Laboratory analysis

Different microbiological tests were used to confirm or rule out measles depending on timing of sampling and sample type. Measles-specific IgM was measured quantitatively in blood (Enzygnost© Siemens) and/or oral fluid (Measles EIA MicroImmune). Detection of measles virus nucleic acid was by in-house quantitative polymerase chain reaction (qPCR) performed on oral fluids, naso-pharyngeal swabs and, in the presence of neurological symptoms, on cerebro-spinal fluid. First-line tests were done by various Belgian laboratories (quality assurance programme accredited by the national body BELAC). If first-line testing was positive (serology and/or PCR), confirmatory testing and genotyping were done by the WHO-accredited National Reference Centre (NRC Measles, Mumps and Rubella, Service of Viral Diseases, Sciensano, Brussels). As per WHO outbreak investigation guidelines, samples from clinical cases with a clear epidemiological link were not sent for laboratory confirmation [11]. Sequenced cases were uploaded by NRC into WHO Measles Nucleotide Surveillance and checked to identify identical sequences circulating.

### Epidemiological investigation

Health officials of AViQ collected demographic data, data on clinical presentation, vaccination status (written documentation of the number of doses required), travel history and info on complications (where present). If infection occurred within the healthcare system (e.g. other patients in the waiting room, staff in the reception area) this was considered nosocomial transmission. Clinicians were asked to report both a yes/no answer to the question ‘clinical criteria satisfied?’ as well as to list the symptoms that were present. Whenever there were discrepancies between those two answers a conservative approach was taken, assuming clinical criteria to be met. Incomplete forms were returned for completion once. Data on ethnicity was not collected but some info on nationality was available. For hospitalised cases, defined as cases with at least one overnight stay at the hospital, more detailed data on measles-related complications and outcome was sought through consultation of treating physicians and hygiene professionals involved in patient-care.

### Statistical analysis

Data were analysed by the national public health institute Sciensano. The Fisher's exact test was applied to compare proportions in 2 × 2 tables, the Freeman–Halton test for tables larger than 2 × 2 and the Mann–Whitney–Wilcoxon test for comparing medians. Multivariable logistic regression was used to assess risk factors for hospitalisation, with age (three age groups), vaccination (at least one dose *vs.* unvaccinated) and gender as exposures. A sensitivity analysis was done using the same multivariable model but assuming all patients with unknown hospitalisation status were not hospitalised.

All analyses were performed using SAS® Enterprise Guide v7.1 (SAS Institute Inc., USA) and R 3.4.4. Excel spreadsheets were used to collect data and produce graphs.

## Results

### Outbreak description

A total of 289 cases of measles were identified between 20 December 2016 and 11 May 2017. Amongst them, 182 (63%) were confirmed by laboratory testing, 78 (27%) were classified as probable cases (epidemiological link with a laboratory confirmed case) and 29 (10%) were possible cases based on clinical symptoms only. All confirmed cases were infected by a genotype B3 virus. No measles-related deaths were reported. Table 1 summarises the demographic characteristics, vaccination status and clinical characteristics of measles cases. Data are presented as both overall numbers and subdivided by hospitalisation status (hospitalised *n* = 125; not hospitalised *n* = 95 and hospitalisation unknown *n* = 69).

**Table 1.** Characteristics of measles cases by hospitalisation status during outbreak in Wallonia, Belgium, Dec 2016–May 2017

	All cases (% of total) (*n* = 289)	Hospitalised (% hospitalised) (*n* = 125)	Not hospitalised (% of not-hospitalised) (*n* = 95)	*P*-Value* Fisher's exact univariate	*P*-Value* multivariable regression
**Classification of case**					
Possible^a^	29 (10%)	0 (0%)	14 (15%)		/
Probable	78 (27%)	22 (18%)	24 (25%)		
Confirmed	182 (63%)	103 (82%)	57 (60%)		
**Gender**					
Male	145 (50%)	66 (53%)	44 (46%)		
Female	144 (50%)	59 (47%)	51 (54%)	0.42	0.10
**Age group (pragmatic)**					
<12 months	33 (11%)	17 (14%)	10 (11%)	0.54^b^	
1–14 years	104 (36%)	35 (28%)	39 (41%)	0.05^b^	
⩾15 years	152 (53%)	73 (58%)	46 (48%)	0.17^b^	0.045
**Age group (detailed)**					
<1 year	33 (11%)	17 (14%)	10 (11%)	0.54^b^	/
1–4 years	48 (17%)	20 (16%)	18 (19%)	0.60^b^	
5–14 years	56 (19%)	15 (12%)	21 (22%)	0.06^b^	
15–29 years	80 (28%)	39 (31%)	21 (22%)	0.17^b^	
⩾30 years	72 (25%)	34 (27%)	25 (26%)	1.00^b^	
**Vaccination status**					
Unvaccinated	117 (40%)	73 (58%)	37 (39%)		
1 dose	19 (7%)	5 (4%)	13 (14%)		
2 doses	7 (2%)	1 (1%)	5 (5%)		
Unknown number of doses	13 (4%)	4 (3%)	9 (9%)	<0.01**	<0.01**
Unknown status	133 (46%)	42 (34%)	31 (33%)		
**Clinical symptoms**					
Meeting clinical criteria^a^	167 (58%)	92 (74%)	72 (76%)		
Classical triad^a^ NOT present	44 (15%)	24 (19%)	16 (17%)	0.72	
Unknown	78 (27%)	9 (7%)	7 (7%)		
**Complications**					
None	90 (31%)	17 (14%)	55 (58%)		
Minimum one	113 (39%)	102 (82%)	11 (12%)	<0.01	
Unknown	86 (30%)	6 (5%)	29 (31%)		

* Comparing hospitalised *vs.* not-hospitalised.

** Comparing unvaccinated *vs.* at least one dose.

a As defined by ECDC: fever + rash + any of cough/coryza/conjunctivitis.

b Comparing one age group with all others.

**Fig. 1.**
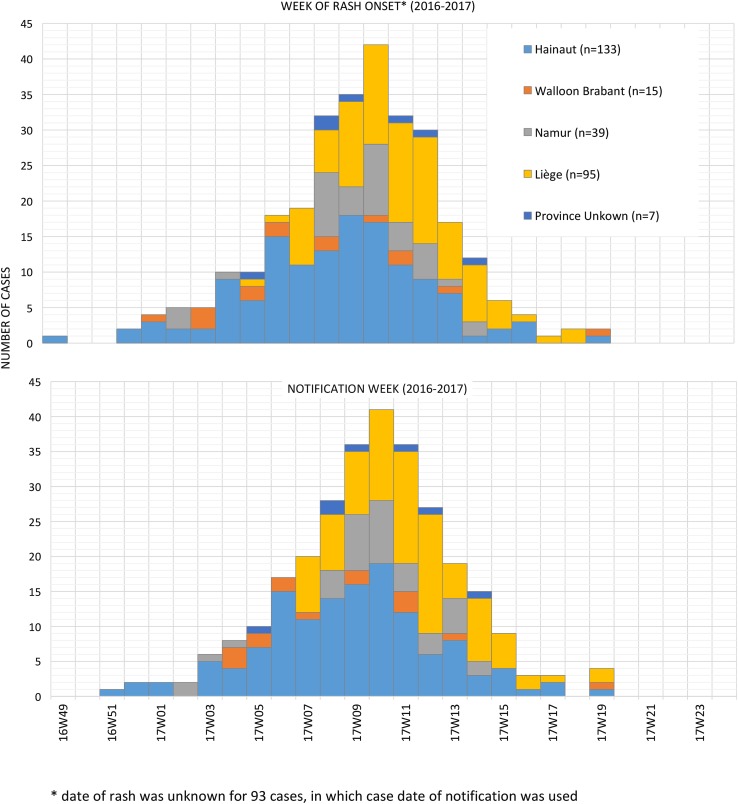
Number of measles cases per week and province by time of rash onset (upper graph) and time of notification (lower graph) during outbreak in Wallonia, Belgium, 12 December 2016 (week 49)–11 May 2017 (week 19) (*N* = 289).

All 289 cases occurred in the Wallonia region of Belgium. The outbreak initiated in week 49 of 2016 in the province of Hainaut, which saw overall the highest number of cases (133). Cases were also notified in the provinces of Liège (95 cases), Namur (39 cases) and Brabant-Wallon (15 cases). The last cases were notified in week 19 of 2017. Figure 1 illustrates the number of cases per week and province by time of rash onset (if known) or time of notification.

Of all patients, 36% visited the emergency department (105/289). Thirty-seven (12.8%) measles cases were reported being contracted in a hospital environment. Spread occurred from patient-to-patient, but also between patients and staff (both directions) and amongst staff. Only one infection was known to be contracted in a day nursery and one in a school. For 73 (25.3%) cases a clear family-link was reported. For the other cases no specific information was available.

### Demographic characteristics, vaccination status and clinical characteristics

Median age was 16 years (range 0–68 years), with 33 cases <1 year (11%), 104 cases between 1–14 years (36%) and 152 of ⩾15 years (53%). Incidence was highest in infants <1 (88.3 cases/100 000 person-years). Five children were younger than 6 months. Five infections occurred abroad, of which three in Romania. The Romanian cases further spread to 51 cases, many of whom were of Eastern European origin.

**Fig. 2.**
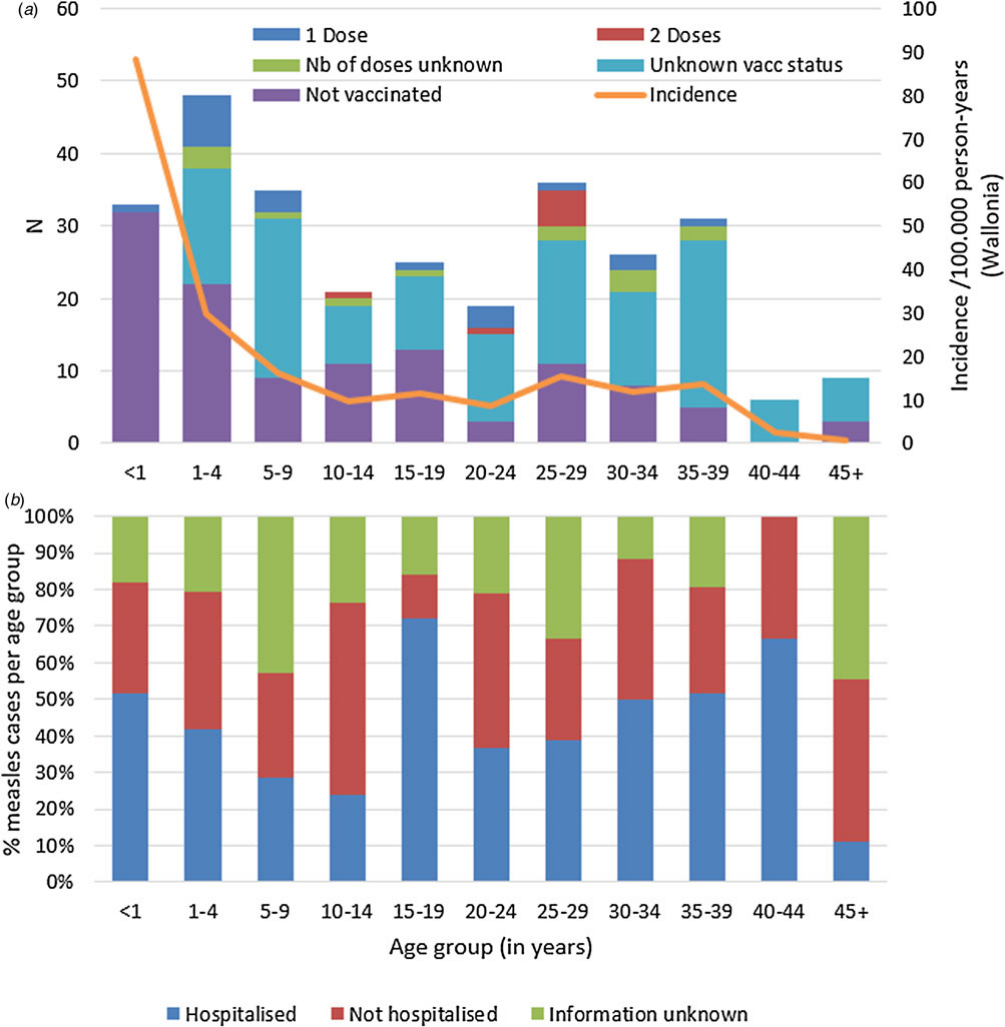
Measles cases during outbreak in Wallonia, Belgium, subdivided by age group, and (a) by vaccination status and incidence per age group, and (b) proportion (in %) requiring hospitalisation (*N* = 289).

According to Belgium's vaccination programmes, the MMR1 is given at 12 months and MMR2 at age 10–12. Table 2 shows the number of cases by (recommended and true) vaccination status and age. Only 57 cases (20%) had proof of the recommended number of vaccinations for their age. Hundred-seventeen cases (41%) were not vaccinated, although 83 of them (29% of total cases) should have received at least one dose in the routine vaccination programmes. Vaccination status was unknown for 133 cases (46%) despite investigation. Figure 2a illustrates number of cases and incidence by age group and vaccination status.

**Table 2.** True vaccination status of measles cases compared to Belgian guidelines

	Unvaccinated	1 dose	2 doses	Number of doses unknown	Unknown	Total (*n* = 289) (% of total)	% vaccinated as recommended
<12 months	32 (27%)	1 (5%)^a^	0 (0%)	0 (0%)	0 (0%)	33 (11%)	100%
1–12 years	35 (30%)	10 (53%)	0 (0%)	5 (38%)	44 (33%)	94 (33%)	11%
>12 years	48 (41%)	8 (42%)	7 (100%)	8 (62%)	84 (63%)	155 (54%)	5%
<1970	2 (2%)	0 (0%)	0 (0%)	0 (0%)	5 (4%)	7 (2%)	100%
	117 (100%)	19 (100%)	7 (100%)	13 (100%)	133 (100%)	289 (100%)	57 (20%)

Cases who received the recommended number of doses for the age group (per Belgian guidelines) are shaded. People <1970 are considered immune in Belgium.

a An advanced dose may be recommended under special circumstances, e.g. in case of travel to endemic area.

Detailed information on clinical symptoms was available for 211 cases (73%) and is presented in Table 3. A total of 44 cases (21% of 211), of which 33 were lab-confirmed, did not present the classical triad (fever, rash and any of cough, coryza or conjunctivitis). Only six of these did *not* present with rash; 33 cases had both fever and rash, four cases presented with rash only and one case had rash, cough and coryza but no fever. In 12 cases, rash appeared after the time of notification. The proportion of non-classical presentations *vs.* classical presentations did not differ between age groups (*P* = 0.33). There was a trend towards more non-classical presentations in vaccinated patients, although not significant at the 0.05-level; half of all fully vaccinated patients presented without the triad (3/6, 50%), *vs.* one quarter of those who had received 1 dose (5/19, 26%) and only one out of six of the unvaccinated (17/109, 16%, *P* = 0.08). Non-classical presentations were equally frequent in both hospitalised and non-hospitalised cases (24/116 = 21% *vs.* 16/88 = 18% *P* = 0.72). Four patients with non-classical symptoms were index patients of smaller clusters and contaminated a further 11 persons. Vaccination status for these index patients was unknown for two and unvaccinated for the other two.

**Table 3.** Number of non-classical presentations by age group, vaccination and hospitalisation status during a measles outbreak in Wallonia, Belgium, Dec 2016–May 2017

	Non-classical (*n* = 44)	Classical (*n* = 167)	No info (*n* = 78)	Total (*n* = 289)	*P*-Value*
**Age group**					
<12 months	5	22	6	33 (11%)	
1–4 years	8	30	10	48 (17%)	
5–14 years	10	23	23	56 (19%)	
15–29 years	13	44	23	80 (28%)	
⩾30 years	8	48	16	72 (25%)	0.48
**Gender**					
Male	19	85	41	145 (50%)	
Female	25	82	37	144 (50%)	0.40
**Hospitalisation**					
Hospitalised	24	92	9	125 (43%)	
Non-hospitalised	16	72	7	95 (33%)	0.72
Unknown	4	3	62	69 (24%)	
**Vaccination status**					
Unvaccinated	17	92	8	117 (40%)	
1 dose	5	14	0	19 (7%)	
2 doses	3	3	1	7 (2%)	0.08**
Unknown number of doses	2	11	0	13 (5%)	
Unknown status	17	47	69	133 (46%)	

* Based on Fisher's-exact comparing non-classical *vs.* classical.

** Comparing unvaccinated *vs.* one or two doses.

Five cases were pregnant, of which four were hospitalised (no data for the fifth woman). Reported complications in pregnancy were dehydration/diarrhoea (*n* = 2), pneumonia (*n* = 1) and hepatic disorders (*n* = 2). Delivery had to be induced at 35 weeks for one woman with severe pneumonia. Diagnosis was delayed several days for two of the pregnant women who were hospitalised in the maternity ward (differential diagnoses Epstein-Barr infection and intrahepatic cholestasis of pregnancy).

### Cases in health care workers

Thirty-six (12%) of the cases were health care workers (HCW). Among them, 29 (81%) were involved in direct patient care, including two healthcare students. Age of staff varied between 22 and 57 years, with a median age of 30 years. Vaccination status was unknown for 17 HCWs (47%), not vaccinated for 6 (17%), vaccinated with an unknown number of doses for 6 (17%), vaccinated with one dose for 2 (6%) and with two doses for 5 (14%). Three unvaccinated HCWs and 10 with unknown vaccination status were born before the start of the immunisation programmes (1985). At least 17/36 HCWs got infected in their workplace (incomplete data, missing for 17/36 and known family link for 2/36). HCWs were found to be the source of infection for three patients.

### Hospitalised cases

Data on hospitalisation-status were available for 220 out of the 289 cases (76%). Overall, 125 (43%) of all measles cases were known to be hospitalised. Distribution of hospitalisation status per age group during the outbreak is shown in Figure 2b. The highest hospitalisation rates were seen in the group of 15–19 years and in the 40–44 year olds (18/2572.0% and 4/6, 66.7%, respectively). The lowest hospitalisation rate is in children aged 5–14 with 26.8% (15/56). Table 4 summarises additional data on hospitalised cases, where available. All hospitalised cases were probable or confirmed cases.

**Table 4.** Characteristics of hospitalised measles cases by age group (*n* = 125)

	Total (*n* = 125)	<1 year (*n* = 17)	1–4 years (*n* = 20)	5–14 years (*n* = 15)	15–29 years (*n* = 39)	30+ (*n* = 34)
**Min–Max length of stay** (in days) (IQR)	1;42 (3;7)	2;13 (4;7)	1;8 (3;5)	1;9 (3;5)	1;42 (3;7)	1;13 (4;7)
**Min–Max time to notification** (in days) (IQR)^a^	−18;22 (−1;0)	−3;2 (0;1)	0;5 (0;2)	−18;10 (0;1)	−5;16 (0;1)	−3;22 (0;3)
**Complications**^b^ ***n*** (% of age group)						
Hepatic disorders	58 (49%)	5 (29%)	6 (30%)	6 (40%)	20 (51%)	21 (62%)
Dehydration/diarrhoea	36 (30%)	3 (18%)	10 (50%)	6 (40%)	10 (26%)	7 (21%)
Pneumonia	19 (16%)	3 (18%)	2 (10%)	1 (7%)	5 (13%)	8 (24%)
Bronchitis/respiratory problems	5 (4%)	1 (6%)	0 (0%)	0 (0%)	3 (8%)	1 (3%)
Otitis media	5 (4%)	3 (18%)	1 (5%)	1 (7%)	0 (0%)	0 (0%)
Acute encephalitis	3 (3%)	0 (0%)	0 (0%)	0 (0%)	3 (8%)	0 (0%)
Renal insufficiency	4 (3%)	1 (6%)	0 (0%)	0 (0%)	1 (3%)	2 (6%)
Pancreatitis	1 (1%)	0 (0%)	0 (0%)	0 (0%)	1 (3%)	0 (0%)
Rhabdomyolysis	1 (1%)	0 (0%)	0 (0%)	0 (0%)	1 (3%)	0 (0%)
Stomatitis/Herpangina	10 (8%)	1 (6%)	2 (10%)	0 (0%)	2 (5%)	5 (15%)
Other	61 (51%)	8 (48%)	13 (65%)	7 (47%)	16 (41%)	17 (50%)

a A negative result means that notification was done before date of hospitalisation. Zero means that notification was done on the day of hospitalisation.

b Multiple complications per patient possible.

For further analysis, we used three pragmatic age groups: infants (<12 months), children (1–14 years) and adolescents/adults (15 years and above). In multivariable analysis of age (in three groups), gender and vaccination status (unvaccinated *vs.* at least one dose) as risk factors for hospitalisation, unvaccinated status (OR 6.0 (2.8–17.1) *P* < 0.01) and age ⩾15 years (OR 2.3 (1.03–5.24) *P* = 0.045) were associated with hospitalisation. In this model, thus correcting for vaccination status and gender, children under one were not found to be more likely to be hospitalised (OR 1.2 (0.5–3.4)). As information on hospitalisation was missing for 69 cases (24%), we performed a sensitivity analysis assuming all cases with missing hospitalisation status to be not hospitalised. The effect of older age and unvaccinated status remained essentially unchanged, but an association with male gender was now also found (OR 2.20 (1.1–4.57) *P* = 0.04).

Median length of stay was 5 days, ranging from 1 to 42 days. Hospitalisations were significantly shorter for 1–14 year olds compared to the other two age groups (*P* = 0.02). For 16 cases (13%) measles was suspected and notified to health authorities prior to hospital admission, for 64 cases (51%) notification was made within 24 h of admission and for eight cases (6.4%) ⩾5 days after hospital admission. Time to notification (in days from day of hospital admission) was not significantly different between the age groups and depending on presentation with/without typical triad.

Overall, hepatic disorders (cytolysis and/or cholestasis) were the most commonly observed complication (58 cases), followed by dehydration and/or diarrhoea (36 cases), and pneumonia (19 cases). Three cases of acute encephalitis were reported and five had otitis media.

**Fig. 3.**
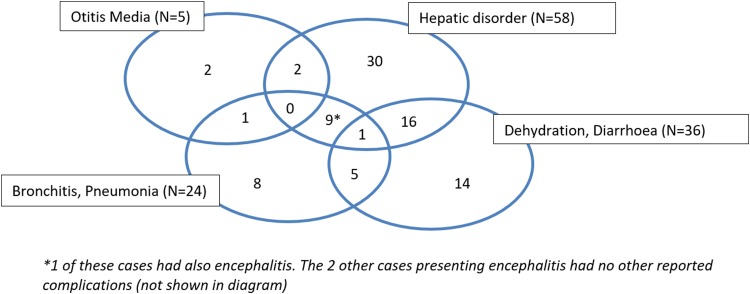
Venn diagram of measles-associated complications in hospitalised cases during outbreak in Wallonia, Belgium, 20 Dec 2016–11 May 2017 (*N* = 119).

The proportion of cases with pneumonia was not significantly different between the three age groups. Otitis media was only observed in children <15 years. Almost half (16/35 = 46%) of all children between 1–14 years presented with dehydration and/or diarrhoea compared to less than 25% in the other two age groups (*P* = 0.03 for 1–14 years *vs.* <12 months and *P* = 0.02 for 1–14 years *vs.* ⩾15 years). Hepatic disorders were noted in all age groups, but occurred significantly more often in those aged 15 and above (56%, 41/73) *vs.* children between 1–14 years (34%, 12/35,) or infants under one (29.4%, 5/17, *P* = 0.03 comparing three groups). Rare severe complications such as acute encephalitis, rhabdomyolysis and pancreatitis were reported solely in patients aged 15 years and older. Thirty-four patients had several measles-associated complications simultaneously. Figure 3 illustrates the overlap of complications in patients with regard to five main complication categories: otitis, respiratory problems, dehydration and/or diarrhoea, hepatic disorders and encephalitis.

Ninety-three hospitalised patients (74%) were admitted through emergency services. Cases were subsequently mostly hospitalised in the paediatric ward (40%) but also in a variety of other units, including units with vulnerable patients like the obstetric ward (2%).

Five patients had to be admitted to intensive care units (ICU) during their stay. Reasons for ICU were acute encephalitis with or without organ failure (renal insufficiency, bronchitis, hepatic cytolysis), and pneumonia in combination with other conditions (hepatic cytolysis, pregnancy, renal insufficiency). Length of stay in ICU ranged from 1 to 18 days. All patients were young adults, between 26 and 37 years of age. They all recovered, although the presence of long-term sequelae is unknown.

## Discussion

In this article, we provide a more in-depth analysis of a measles outbreak in Wallonia, Belgium, affecting 289 people. Measles has been targeted by the WHO for elimination in Europe by 2020 but recent years have seen an increase in the number of countries reporting outbreaks [8].

The epidemiology of measles has changed after the widespread introduction of the vaccine and it is no longer a typical childhood disease [8 12 13]. In this outbreak we saw cases in all age groups, the oldest affected person being 68 years old, born in 1948. Coverage of MMR1 at ages 18–24 months in Wallonia has been reported to have increased from 82.4% (1999) to as high as 95.6% in 2015 [14]. However, many susceptible individuals remain due to the historically low vaccination coverage. Moreover, people born between 1970–1984 are vulnerable, as they were not yet offered the vaccine in routine programmes, but might have been insufficiently exposed to naturally circulating measles during their childhood. A total of 117 patients (40%) had not received any vaccination, despite the fact that 83 of them should have been included in routine vaccination programmes (71% of unvaccinated cases, 29% of total cases). Additionally, vaccination status was unknown for 133 (46%) of cases. These figures underline the importance of adherence to guidelines and documentation of vaccination status. If the elimination goals are to be reached, it is crucial that catch-up campaigns are implemented.

In this outbreak, no deaths were reported yet 43% of all patients required hospitalisation. This is high in comparison with previously reported figures [4 15 16] but identical to the percentage of hospitalised patients in the 2017 outbreak in Italy [17]. In multivariable analysis, hospitalisation rates were significantly higher in patients ⩾15 years but not in infants <1 year. In contrast, previous literature has described higher rates of hospitalisations in both the younger and older patients [4 6 18 19]. The difference with published literature might be explained by the fact that we corrected for vaccination status. Moreover, at the time of the outbreak there was little experience with measles and both parents and doctors might have felt safer hospitalising children, even if the cases were relatively mild. Hospitalisations were significantly shorter for 1–14 year olds compared to the other two age groups (*P* = 0.02). The type of complications in hospitalised patients also differed according to age. Otitis media was exclusively observed in younger patients (<15 years) and dehydration/diarrhoea was markedly more frequent in children (48% 1–14 years) compared to adolescents/adults (25%) and infants (18%). Rare and severe complications such as encephalitis and pancreatitis were observed only in patients over 15 years. Similarly, all five patients that required intensive care were young adults (26–37 years), comparable to the 36 ICU cases during the 2011 outbreak in France which had a median age of 29.2 years (interquartile range 27.2–34.2) [20]. Hepatitis in adults with measles (present in 59% of hospitalised cases ⩾15 years) has been long described [21 22] but may be misleading when it is the predominant sign upon presentation. The clinical significance of hepatitis in measles and whether it should be assumed to be a typical disease manifestation, rather than a complication, remains debated [23 24]. Unfortunately, we do not have additional information on severity of hepatocellular injury or clinical relevance, having only information on the presence or absence of abnormal liver biochemical tests.

Fifteen percent of all cases did not meet the ECDC clinical criteria, i.e. they did not present with the classical triad of fever, rash and at least one of either cough, coryza or conjunctivitis [1]. Whilst a highly specific case-definition is needed in order to allow for standardised international reporting and comparison, thereby avoiding false-positives, a too strict (application of a) definition can lead to missed cases. Missing cases does not only bias statistics, more importantly it can impact diagnosis and treatment of the individual cases and delay implementation of outbreak control measures. This is especially important in fully vaccinated persons, as it is known that approximately three percent of fully vaccinated people can still suffer from measles, but cases will be attenuated and milder [4 25 26]. A combination of rash and fever should prompt laboratory testing to confirm or rule out measles. Especially rash seems to be a very sensitive symptom (absent in only 6 out of 211 cases).

Early isolation of suspected cases is imperative, but hampered by the non-classical presentations. Furthermore, the outbreak coincided with the seasonal peak of Influenza in Belgium (week 1–9 in 2017), which increased the potential for misdiagnosis and meant healthcare systems were experiencing a very high workload. If cases are not promptly identified and isolated within healthcare settings, their presence in the waiting room can endanger other patients with weakened immune systems. Moreover, the high number of potential contacts means a huge workload for public health authorities doing contact tracing. Delayed identification in combination with the highly contagious nature of measles and a high number of patients presenting at the emergency department (36% of all cases), made nosocomial spread an important factor in this outbreak. This has also been previously described in several other outbreaks [17 27 28].

Potential for nosocomial spread is further increased by poorly vaccinated HCWs (including support personnel working in the hospital) [27 29 30]. The variety of wards were measles cases were hospitalised again points out the need of protection against measles for all staff on the hospital floor, not only in perceived ‘high-risk’ areas like the emergency or paediatric department. Despite the fact that HCWs were identified as a priority group for catch-up vaccinations in the Action Plan 2016–2020 by the Committee for the Elimination of Measles and Rubella in Belgium [31], only 5/36 involved HCWs had documentation of full vaccination.

Challenges in outbreak management were the previously mentioned diagnostic challenges, suboptimal vaccination coverage and poor documentation of vaccination status. Currently, a system of electronic record keeping of administered vaccines is being rolled out in Wallonia (www.e-vax.be). Efforts are being made to improve vaccination coverage amongst HCWs, mainly by reiterating the message to occupational health physicians and decrease financial barriers, rather than by making vaccination of HCWs mandatory. At a minimum, vaccination records should be completed and readily available for all hospital staff. Public health messages distributed through the general media are meant to raise awareness about measles, thereby both facilitating early diagnosis and improving vaccination coverage. However, their success will likely be higher if catch-up vaccines are made free-of-charge for everyone born after 1970. In order to shorten the interval between doses and increase MMR2 coverage, the Belgian Superior Health Council decided in March 2019 to bring down the recommended age for administering MMR2 to 7–9 years (instead of 10–12 years previously) in order to shorten the interval between doses and increase MMR2 coverage. This should help prevent cases in the 1–12 year group, which represented 94 cases (33%) in the present outbreak.

Additional challenges in this outbreak consisted of logistic difficulties in implementing post-exposure prophylaxis. Although prophylactic vaccination was recommended to all persons who had contact with a measles case less than 72 h before and were not considered immune, authorities were relying on patients presenting to their general practitioners to administer the vaccines. Both the consultation and the vaccine (when used outside routine immunisation campaigns) need to be paid for out-of-pocket, which may decrease acceptance and uptake. It is unknown how many of the traced contacts did indeed receive the vaccine. Mobile vaccination teams like the ones already existing in the northern region of Belgium, Flanders, clearly have an important role in decreasing barriers to access and should be founded in Wallonia too. The mobile team in Flanders previously reported issues with mistrust and language barriers when reaching out to the Roma community [32]. These issues are likely to have played a role in this outbreak too.

This outbreak analysis allows us to learn about real-life clinical and logistical challenges during a measles outbreak. There are however some limitations to bear in mind. At the height of an epidemic, information is mainly collected for outbreak containment purposes and obtained clinical information is less detailed. However, we strived to make data collection as complete as possible for hospitalised cases by direct collaboration with hospitals and physicians. Twenty hospitals in the region were contacted, so cases with missing data on hospitalisation (*n* = 69, 24%) can be assumed to have been treated as outpatients. Nevertheless, as the description and analysis in this article stem from initial epidemiological investigations with a public health objective, individual clinical data on e.g. pre-existing conditions (except for pregnancy) or symptomatic hepatic disorder (as opposed to purely biochemical abnormalities) were not available. For 11 of the non-classical cases laboratory confirmation was not sought and we rely on the physician's expertise (high clinical suspicion in combination with epidemiological link) for their classification as non-classical measles cases. Therefore some of these cases might have been misclassified. Clinical symptoms should have been taken into account for the classification of the cases regardless of when they developed (e.g. date of rash is known to have been after date of notification for 12 cases), but it is possible that some symptoms were underreported if they developed later, which would lead to an overestimation of the number of atypical presentations.

In conclusion, since widespread introduction of a highly-effective vaccine, measles no longer presents as a typical childhood disease. In this outbreak, more than half of all patients were over 15 years of age and rate of hospitalisation was over 40%. Clinicians must therefore be aware of the specificities of adult measles to allow for early diagnosis and correct management. As observed in the present outbreak, this includes more non-classical presentations and a different spectrum of complications and severity. Timely diagnosis is key to allow public health authorities to implement preventive measures. If the elimination target is to be reached, attention should be paid to free of charge catch-up vaccination programmes, availability of vaccination records, immunity status of HCWs and isolation protocols in hospitals.

## References

[1] European Commission (2018) Commission Implementing Decision (EU) 2018/945 of 22 June 2018 on the Communicable Diseases and Related Special Health Issues to be Covered by Epidemiological Surveillance as Well as Relevant Case Definitions. Luxembourg: European Commission.

[2] European Centres for Disease Prevention and Control. Factsheet About Measles. European Centres for Disease Prevention and Control Stockholm. Available at https://www.ecdc.europa.eu/en/measles/facts/factsheet (Accessed 12 Nov 2019).

[3] Centres for Disease Control (2015) Pinkbook | Measles | Epidemiology of Vaccine Preventable Diseases, 13th edn. Washington D.C: Centres for Disease Control, pp. 209–230. Available at https://www.cdc.gov/vaccines/pubs/pinkbook/meas.html%complications (Accessed 13 November 2019).

[4] Centres for Disease Control and Prevention, National Center for Immunization and Respiratory Diseases D of VD. Measels Vaccination. Atlanta: Centres for Disease Control and Prevention, National Center for Immunization and Respiratory Diseases D of VD Available at https://www.cdc.gov/measles/vaccination.html (Accessed 13 November 2019).

[5] Grammens T (2018) Couverture Vaccinale en Belgique [Vaccination Coverage in Belgium]. Brussels: Sciensano.

[6] World Health Organization(2019) Measles Fact Sheet. News Room. Available at https://www.who.int/news-room/fact-sheets/detail/measles (Accessed 11 June 2019).

[7] World Health Organization (2012) Global Measles and Rubella Strategic Plan 2012–2020. Geneva: World Health Organization.

[8] European Centre for Disease Prevention and Control (2018) Measles and Rubella Surveillance-2017. Stockholm: European Centre for Disease Prevention and Control.

[9] Grammens T (2016) Different measles outbreaks in Belgium, January to June 2016 - a challenge for public health. Euro Surveillance 21, 303-13. 10.2807/1560-7917.ES.2016.21.32.30313 PMC4998501 27541858

[10] Grammens T (2017) Ongoing measles outbreak in wallonia, Belgium, December 2016 to march 2017: characteristics and challenges. Eurosurveillance 22. 10.2807/1560-7917.ES.2017.22.17.30524 PMC5434888 28488998

[11] European Office of the World Health Organization (2013) Guidelines for Measles and Rubella Outbreak Investigation and Response in the WHO European Region. Copenhagen: European Office of the World Health Organization.

[12] Ma R (2017) An expensive adult measles outbreak and response in office buildings during the era of accelerated measles elimination, Beijing, China. Vaccine 35, 1117–1123. 28131395 10.1016/j.vaccine.2017.01.021

[13] European Centre for Disease Prevention and Control (2019) Rapid Risk Assessment - Who is at Risk fo Measles in the EU/EEA? May 2019. Stockholm: European Centre for Disease Prevention and Control.

[14] Swennen B (2016) Vaccination Coverage in the Wallonia-Brussels Federation in 2015 (Couvertures Vaccinales en Fédération Wallonie-Bruxelles en 2015). Provac, Brussels.

[15] Moss WJ and Griffin DE (2012) Measles. Lancet 379, 153–164. 21855993 10.1016/S0140-6736(10)62352-5

[16] European Centre for Disease Prevention and Control (2018) Risk of Measles Transmission in the EU / EEA Main Conclusions and Options for Response. Stockholm: European Centre for Disease Prevention and Control.

[17] Filia A (2017) Ongoing outbreak with well over 4,000 measles cases in Italy from January to end August 2017 – what is making elimination so difficult? Eurosurveillance 22. 10.2807/1560-7917.ES.2017.22.37.30614 PMC5607657 28933342

[18] Perry RT and Halsey NA (2004) The clinical significance of measles: a review. Journal of Infectious Diseases 189, S4–16. 15106083 10.1086/377712

[19] Muscat M EUVAC.NET Group (2009) Measles in Europe: an epidemiological assessment. Lancet 373, 383–389. 19131097 10.1016/S0140-6736(08)61849-8

[20] Rafat C (2013) Severe measles infection: the Spectrum of disease in 36 critically Ill adult patients. Medicine 92, 257–272. 23982057 10.1097/MD.0b013e3182a713c2 PMC4553975

[21] Tishler M and Abramov AL (1983) Liver involvement in measles infection of young adults. Israel Journal of Medical Sciences 19, 791–793. 6643015

[22] Gavish D (1983) Hepatitis and jaundice associated with measles in young adults. An analysis of 65 cases. Archives of Internal Medicine 143, 674–677. 6838292

[23] Dinh A, Fleuret V and Hanslik T (2013) Liver involvement in adults with measles. International Journal of Infectious Diseases 17, e1243–4. 23938044 10.1016/j.ijid.2013.06.014

[24] Merino E (2014) Measles in adults during an outbreak in Spain: hospitalization associated with gastrointestinal and liver involvement. Infection 42, 763–765. 24962127 10.1007/s15010-014-0650-0

[25] Artimos de Oliveira S (2000) Atypical measles in a patient twice vaccinated against measles: transmission from an unvaccinated household contact. Vaccine 19, 1093–1096. 11137243 10.1016/s0264-410x(00)00338-8

[26] World Health Organization. Manual for the laboratory-based surveillance of measles, rubella, and congenital rubella syndrome In Manual for the Laboratory-Based Surveillance of Measles, Rubella, and Congenital Rubella Syndrome, 3rd edn. Geneva, Switzerland Available at: https://www.who.int/immunization/monitoring_surveillance/burden/laboratory/manual_section8.5/en/

[27] Porretta A (2017) A nosocomial measles outbreak in Italy, February-April 2017. Eurosurveillance 22. 10.2807/1560-7917.ES.2017.22.33.30597 PMC5572940 28840827

[28] Botelho-Nevers E (2012) Nosocomial transmission of measles: an updated review. Vaccine 30, 3996–4001. 22521843 10.1016/j.vaccine.2012.04.023

[29] Maltezou HC and Wicker S (2013) Measles in health-care settings. American Journal of Infection Control 41, 661–663. 23352075 10.1016/j.ajic.2012.09.017

[30] Botelho-Nevers E (2011) Measles among healthcare workers: a potential for nosocomial outbreaks. Eurosurveillance 16. 21251488

[31] Committee for the Elimination of Measles and Rubella in Belgium. [Action Plan 2016–2020] [Dutch]. Brussels.

[32] Van De Mieroop E Acitivies Report Mobile Vaccination Team Period 1/4/2015–31/3/2017 [Activiteitenverslag: Mobiel Vaccinatieteam Periode 1/4/2015–31/3/2017] [Dutch].

